# Efficacy of Sensor-Based Training Using Exergaming or Virtual Reality in Patients with Chronic Low Back Pain: A Systematic Review

**DOI:** 10.3390/s24196269

**Published:** 2024-09-27

**Authors:** Giovanni Morone, Foivos Papaioannou, Alberto Alberti, Irene Ciancarelli, Mirjam Bonanno, Rocco Salvatore Calabrò

**Affiliations:** 1 Department of Life, Health and Environmental Sciences, University of L’Aquila, 67100 L’Aquila, Italy; giovanni.morone@univaq.it (G.M.); irene.ciancarelli@univaq.it (I.C.); 2 San Raffaele Institute of Sulmona, 67039 Sulmona, Italy; 3 Euleria Health srl Società Benefit, Via delle Zigherane, 4/A, 38068 Rovereto, Italy; fivospapaio@gmail.com (F.P.); a.albertifkt@gmail.com (A.A.); 4 IRCCS Centro Neurolesi “Bonino-Pulejo”, Cda Casazza, S.S. 113, 98124 Messina, Italy; roccos.calabro@irccsme.it

**Keywords:** chronic low back pain, IMU, sensor-based rehabilitation, biofeedback, exergame, virtual reality, exercise therapy

## Abstract

In its chronic and non-specific form, low back pain is experienced by a large percentage of the population; its persistence impacts the quality of life and increases costs to the health care system. In recent years, the scientific literature highlights how treatment based on assessment and functional recovery is effective through IMU technology with biofeedback or exergaming as part of the tools available to assist the evaluation and treatment of these patients, who present not only with symptoms affecting the lumbar spine but often also incorrect postural attitudes. Aim: Evaluate the impact of technology, based on inertial sensors with biofeedback or exergaming, in patients with chronic non-specific low back pain. A systematic review of clinical studies obtained from PubMed, Scopus, Science Direct, and Web of Science databases from 1 January 2016 to 1 July 2024 was conducted, developing the search string based on keywords and combinations of terms with Boolean AND/OR operators; on the retrieved articles were applied inclusion and exclusion criteria. The procedure of publication selection will be represented with the PRISMA diagram, the risk of bias through the RoB scale 2, and methodological validity with the PEDro scale. Eleven articles were included, all RCTs, and most of the publications use technology with exergaming within about 1–2 months. Of the outcomes measured, improvements were reported in pain, disability, and increased function; the neuropsychological sphere related to experiencing the pathology underwent improvements. From the results obtained, the efficacy of using technology based on exergames and inertial sensors, in patients with chronic non-specific low back pain, was increased. Further clinical studies are required to achieve more uniformity in the proposed treatment to create a common guideline for health care providers.

## 1. Introduction

Low back pain is a condition that commonly affects the population [1]. Depending on the underlying cause of the painful condition, it can occur in either specific low back pain or non-specific (NS-LBP) [2]. It is defined as specific when the underlying cause is diagnosable, for example, lumbar stenosis, herniated disk, fractured vertebrae, cauda equina syndrome, or tumors [1,2]. In the condition of NS-LBP, the pathoanatomic cause is unclear, but innervated structures become involved by nociceptive endings rendering lumbar pain less functional. Given the poor correlation between cause/symptoms, specific diagnostic investigations, in the case of NS-LBP, are indicated in case of suspicion of an underlying presence of specific pathology [1]. The diagnosis therefore remains mainly linked to the history, including the triggering event, persistence of pain or behavior over 24 h, and the exclusion of specific causes or the exclusion of possible red flags. There is no specific physical test in the case of NS-LBP or chronic LBP. The physical examination, through several active and passive tests on different musculoskeletal structures, considers parameters such as ROM, body posture, and specific facets of pain [1,2]. Based on the duration of the symptom, it is defined as “acute phase” if the pain persists for a maximum of 6 weeks, “subacute” if it ranges from 6 to 12 weeks, while in the case of persistence of pain beyond 3 months, it is addressed as “chronic” (CLBP)” [3]. In the CNS-LBP condition, posture is inevitably influenced by proprioceptive alterations, resulting in tensions of the postural chains and a malposition of the various segments (i.e., reduction in their mobility and difficulty in repositioning); this leads to the maintenance of wrong postures and the maintenance of this error due to the lack of efficient processing of proprioceptive information. However, it is difficult to identify a single cause for the altered motor pattern, as disability (reduction in quantity/quality of the movement), pain, and personal experience are all linked [3,4]. The epidemiology of NS-LBP within LBP cases in the global population is around 90% [1,2], considering the duration of symptoms, in 15% of LBP cases, there is a persistence of symptoms with the development of CLBP [5]. It can be understood how the costs, resulting from the presence of LBP/NSLBP/CLBP, are high in terms of public health expenditure, arising both from the need for treatment (pharmacological, physiotherapeutic, psychological) but also due to the negative effects on productivity in the workplace due to work absenteeism [1,2,3,6]. Due to its widespread prevalence and the limited effectiveness of long-term treatments, there is increasing interest in complementary non-pharmacological therapies for managing various pain disorders, including CLBP. In recent years, among the different methods of rehabilitation approaches to this condition, the possibility of using technology has become more and more present, both at the diagnostic and therapeutic service levels [7]. With the aim of ‘treating’ a patient with CNS-LBP, various technological devices, like virtual reality (VR), can be employed. VR technologies were primarily created for gaming and entertainment to provide a fully immersive experience in a simulated digital environment [8]. In people with CLBP, VR training has demonstrated efficacy in pain reduction by dividing attention to tasks [9]. Moreover, one of the key benefits of VR is the increased intrinsic motivation to actively participate in the therapeutic process. This is in contrast to conventional methods that may lead to boredom or lack of interest [8,9]. However, VR technologies can be combined with inertial motion sensors, footplates, and reflective markers connected to cameras; this feedback is processed by the software, which reports data on the measured parameters [10]. Overall, these systems are known as motion capture (MoCap) systems, since they allow for the detection of movements. In the literature, two fundamental types of MoCap exist, namely optoelectronic systems and inertial measurement units (IMUs) [10]. In particular, optoelectronic systems are considered the “gold standard”, since they present high reliability and accuracy features when compared to other MoCap technologies [11]. On the other hand, these systems are expensive and time-consuming for long-term setup, which requires specific personnel. Moreover, IMUs are small devices that integrate different multiaxial sensors (e.g., accelerometers, gyroscopes, and magnetometers) to detect movements. These systems present several advantages, such as having a low cost, being wearable, and are easy to use, allowing for the monitoring of movement continuously in supervised (at clinic) and unsupervised (at home) settings. However, the main disadvantage is that IMUs are less accurate and reliable than optoelectronic systems. IMUs estimate the orientation of body segments, which may be likely to cause drifting due to the integration of noisy measurements [10]. In the rehabilitation context, both optoelectronic and IMUs are commonly used to perform an objective and quantitative analysis of movement. How the sensors are used may be linked to the performance of a specific skill. Nevertheless, these systems can be used for sensor-based rehabilitation in combination with exergaming, played in full or semi-immersive VR, where the subject (via an avatar) participates in a game.

This systematic review aims to investigate the efficacy of VR rehabilitation therapy, monitored by sensors, on patients with CNS-LBP.

## 2. Materials and Methods

Systematic research was carried out to investigate the current evidence on sensor-based VR training in patients with NS-CLBP. The results of the included studies were summarized according to the Preferred Reporting Items for Systematic Reviews and Meta-Analyses (PRISMA) guidelines [12]. The protocol was registered in the prospective register of systematic reviews (PROSPERO) under ID CRD42023364033.

### 2.1. PICO Question

We defined our combination of search terms using a PICO (population, intervention, comparison, outcome) model. The population was limited to patients with NS-CLBP; we considered sensor-based VR rehabilitation as the intervention; the comparison was evaluated considering the conventional rehabilitation interventions; and the outcomes included any improvements in motor functions (e.g., increased range of motion) and pain.

### 2.2. Search Strategy and Eligibility Criteria

To perform the systematic review, PubMed, Scopus, Science Direct, and Web of Science databases were searched with the following search strategy: (Inertial Measurement Units (IMU) OR Accelerometer sensors OR Gyroscopes OR Wearable sensors) AND (video game-based exercise OR motor learning OR visual biofeedback OR audio biofeedback OR virtual reality) AND (chronic low back pain or non-specific low back pain). Filters applied were as follows: Clinical Trial and Randomized Control Trial. The search included articles published from 1 January 2016 until 1 July 2024 (see Table 1).

Studies were included if they presented the following inclusion criteria: (i) subjects older than 18 years; (ii) clinical studies (e.g., pilot studies, RCTs, clinical trials); (iii) presence of chronic non-specific low back pain; and iv) use of treatment technology (e.g., an exergame or biofeedback provided by sensor technology). On the other hand, we excluded studies with (i) disease-free subjects with chronic low back pain (CLBP)/NSLBP; (ii) technology not inherent to the purpose of the thesis/passive technology in any case not linked to movement; and (iii) no use of technology. We have excluded case reports, case series, and reviews.

### 2.3. Data Extraction and Critical Appraisal

Two reviewers independently screened the titles and abstracts of records found during the search and carried out data extraction. Full-text articles were reviewed individually if either reviewer deemed them potentially eligible for inclusion. Any disagreements about study selection or data extraction were resolved through discussion, with a third reviewer making the final decision. Titles related to the study’s topic were chosen, and a detailed assessment of each study’s title and abstract was initially conducted. The final selection or rejection of studies was based on the inclusion and exclusion criteria after reviewing the full articles.

### 2.4. Risk of Bias and Study Quality Assessment

Two reviewers evaluated the quality of each article using the PEDro scale [13], which ranges from 0 to 10. This scale helps identify clinical trials and assigns each trial a total PEDro score. The PEDro scale consists of 11 criteria to assess the methodological quality of studies and clinical trials, particularly those involving non-pharmacologic interventions. Each criterion a study meets adds one point to the total score, except for the first criterion, which is not scored, resulting in a final score between 0 and 10.

The risk of bias in randomized controlled trials was evaluated using the revised Cochrane risk of bias tool (RoB 2) [14], which covers the following five domains: (i) bias from the randomization process, (ii) bias due to deviations from intended interventions, (iii) bias from missing outcome data, (iv) bias in outcome measurement, and (v) bias in the selection of reported results. To ensure consistent evaluations and a clear understanding of each criterion, an initial calibration meeting was held. A second meeting followed, where the criteria for each included article were reviewed until consensus on the scores was achieved. If disagreements could not be resolved, a third author was consulted for a final decision.

Non-randomized clinical studies were evaluated using the ROBINS-I tool [15], which is designed to assess the risk of bias in the results of non-randomized studies of interventions. The ROBINS-I tool examines bias in the following seven areas: (1) confounding factors, (2) participant selection, (3) intervention classification, (4) deviations from intended interventions, (5) missing data, (6) outcome measurement, and (7) selection of reported results.

## 3. Results

**Figure 1:**
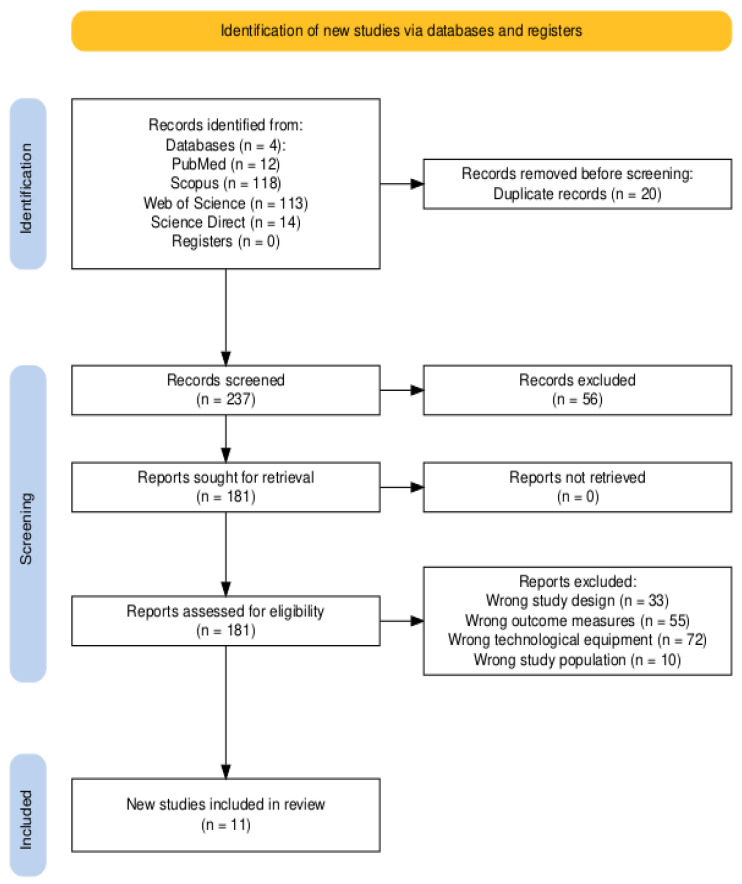
PRISMA flow diagram of the study selection process [12].

A total of 257 articles were found in PubMed, Scopus, Web of Science, and ScienceDirect. After screening for title relevance and duplicates, 20 articles were excluded. An additional 56 articles were excluded due to language, incorrect study aims, incorrect study populations, or inappropriate therapeutic approaches. Consequently, 181 full-text articles were screened, and 11 studies were selected for inclusion in this review (see Figure 1).

We identified 11 studies, comprising eight RCTs [5,16,17,18,19,20,21,22], one cross-over study [23], one feasibility study [24], and one proof-of-concept study [25]. All the included studies dealt with VR therapy with exergames or with specific VR technologies for rehabilitation [25] (i.e., VRRS). In addition, Stamm et al. [21] proposed a multimodal therapeutic approach, combining VR physical therapy with psychoeducation (see Table 2).

### 3.1. Assessment of the Quality of Included Studies—The Risk of Bias

According to the PEDro scale, we found that all articles had an overall good quality score. One article had poor validity [25], seven articles had fair validity [5,17,20,21,22,23,24], two had good validity [16,18], and one article had an excellent validity score [17] (see Table 3). 

**Figure 2:**
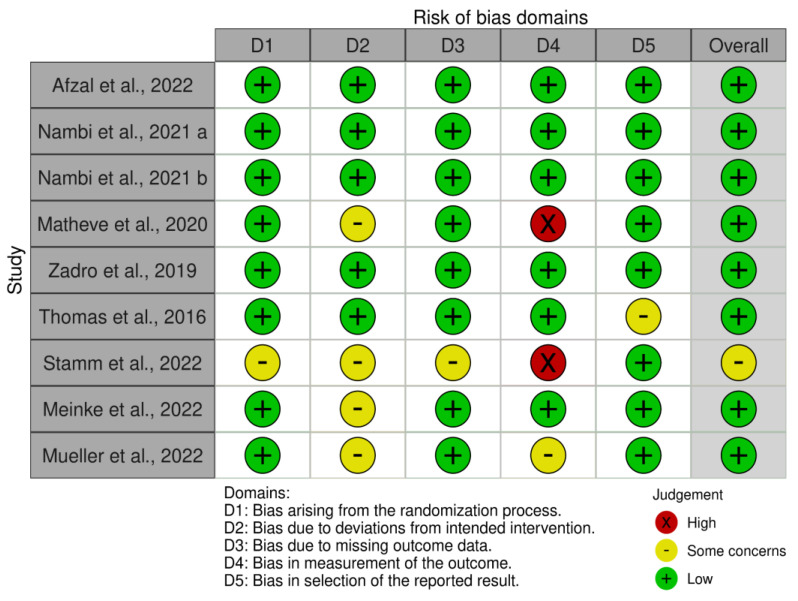
The risk of bias summary evaluated with the RoB tool [5,14,16,17,18,19,20,21,22,23].

Analyzing bias through RoB2 results showed a low risk of bias (see Figure 2). Two publications show few concerns about methodological procedures [12]. Analyzing Graph 2, it can be seen that the included studies show an overall low risk of bias. All articles obtained with the search string are clinical studies, in particular, they are RCTs in which the participants in all groups had CNS-LBP as their basis. In addition, we did not find any biases related to gender or age. Overall, the authors reported a balanced distribution for gender, age, education, and other demographic characteristics, including height, weight, and body mass index. This balance could play a crucial role in reducing selection bias, helping to ensure comparable baseline samples and preventing factors that could compromise the results. The number of participants is varied. Two articles have 84 participants divided into two groups [16,17,18,19]; two studies have 60 participants divided into three groups [18] and two groups [20]; one publication has 56 participants divided into two groups [5]; and one study has 36 participants divided into three groups [17]. Among all the included articles, they utilize exergames as a type of treatment. In the article by Stamm et al. [21], the overall risk of bias has some concerns due to several domains lacking detailed information, particularly regarding the randomization process, allocation concealment, and missing outcome data.

The high risk in the measurement of the outcome domain due to the potential lack of blinding is notable. This is contrary to Meinke et al. [22] and Mueller et al. [23], who both reported an overall low risk of bias. In particular, Meinke et al. [22] demonstrated a low risk of bias, with some concerns primarily due to the lack of participant blinding, which is often challenging to achieve in behavioral interventions. In Mueller et al.’s study [23], a low risk of bias was reported in most domains, with some concerns primarily related to the lack of detailed information on adherence to intervention protocols and blinding of outcome assessors.

**Figure 3:**
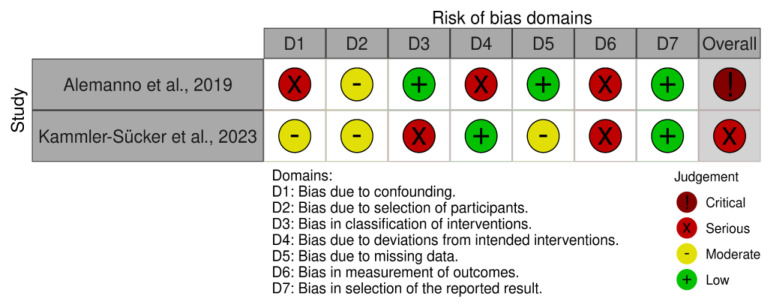
Risk of bias summary evaluated with the ROBINS-I tool [15,24,25].

Non-randomized clinical studies had some serious risk of bias (see Figure 3). Specifically, Alemanno et al. [25] presented a mixture of serious and low risks of bias, with the most significant concerns related to confounding factors, deviations from intended interventions, and the measurement of outcomes. The study was single-armed, with no control group, making it difficult to attribute changes solely to the intervention without accounting for potential external influences. Furthermore, Kammler-Sücker et al. [24] presented a serious risk of bias due to the significant issues in the classification of interventions and the measurement of outcomes. Regarding the latter, outcome measures were well-defined, but there is a risk of bias due to the lack of blinding of outcome assessors, as well as for the classification of the intervention.

### 3.2. Primary Outcomes

In the study by Afzal et al. [16], there were significant improvements in both groups, but with a significantly greater tendency for improvement in the experimental group. In a study by Nambi et al. (a) [17], three outcomes were examined with magnetic resonance imaging (MRI), ultrasound (US), and inflammatory biomarkers. There was significant improvement in the values obtained at the end of treatment (fourth week); the same result was obtained with the control group, but the tendency for improvement was greater for the experimental group, which had significant improvements regarding the Tampa Kinesiophobia Scale (TKS) and the Pain Catastrophizing Scale (PCS). There were significant improvements at the intergroup level regarding pain reduction. In the study by Zadro et al. [20], participants in the experimental group had high levels of PSFS. There was no significant difference between groups in the Pain Self-Efficacy Questionnaire (PSEQ) at post-treatment and after 3 months, while at 6 months, there was a significant improvement for the experimental group compared to the control group. Azfal et al. [16] used a pain and disability assessment. Nambi et al. (a) [17] analyzed the multifidus muscle thickness in the L4-L5 tract using ultrasound. In Nambi et al.’s study (b) [18], the primary outcomes are related to pain and psychosocial status, using the VAS and TSK. The article [10] uses scales related to pain, fear, and disability, namely the Numeric Pain Rating Scale (NPRS), the PCS, the TSK, and the Roland Morris Disability Questionnaire (RMDQ). In the study by Zadro et al. [20], the primary outcomes mainly concern the psychosocial domain assessed with the PSEQ and with the Rapid Assessment for Physical Activity Questionnaire (RAPA). The article [12] uses the following primary outcomes: the visual analogical scale (VAS) and the objective assessment of trunk flexion; the data obtained with flexion are the averages in achieving high-, medium-, and low-height goals. The data were then used to calculate the lowest impact height for levels I°-III° of the game. Stamm et al. [21] evaluated a VR multimodal pain therapy for older adults with CNS-LBP. As primary outcomes, they included clinical scales to evaluate pain (NRS, the Hannover Functional Ability Questionnaire for measuring back pain-related disability—Ffb-H-R, and the Chronic Pain Grade Questionnaire—CPGQ). Regarding pain intensity (NRS), both interventions showed a reduction in pain intensity. However, the reduction was not statistically significant for the VR intervention compared to the control group. Meinke et al. [22] primarily assessed postural balance by the change in anterior–posterior (AP) postural sway between the pre-intervention and post-intervention assessments. However, no significant difference was found in the change in AP sway direction during the intervention period (T2-T3) between the control and intervention groups (W = 99; *p* = 0.36; r = 0.07). Mueller et al. [23] evaluated the maximum angle measurement (the maximum angle of trunk lateral flexion—right or left side—in degrees), without finding significant changes from pre- to post-intervention for any segment (*p* > 0.05). Alemanno et al. [25] evaluated pain (NRS, the McGill Pain Questionnaire—MPQ, and the Brief Pain Inventory—BPI), quality of life (Short Form—SF-36), and cognitive functions through several neuropsychological tests including memory and attention assessments. These authors observed significant reductions in pain scores (NRS, MPQ, BPI) post-treatment (*p* < 0.001). Improvements in QoL across several SF-36 domains were observed, such as physical functioning, bodily pain, vitality, and social role functioning (*p* < 0.05), as well as in cognitive performance, especially in naming, digit span, and Rey figure tests (*p* < 0.05). In addition, Alemanno et al. [25] assessed functional abilities with RMDQ and kinematic data for trunk movements. They found functional improvements were indicated by a significant decrease in RMDQ scores (*p* < 0.001) and enhanced trunk motion range. Overall, 90% of patients reported improvement in pain and quality of life, demonstrating the potential of VR as an effective non-pharmacological treatment for CNS-LBP. Kammler-Sücker et al. [24] measured several outcomes, including pain expectancy (i.e., participants’ expectations of pain before the sessions), ROM in side-bending and rotation in the horizontal plane, engagement due to VR therapy, functional capacity for movements, and the limitation of movement and pain during movements. The authors found that the group that received the avatar-based intervention showed a marginally significantly higher engagement level compared to the control group. However, there were no significant effects on pain during movement, functional capacity, or movement limitations. Prior pain expectancy played a significant role in influencing self-reports on pain and function (see Table 4).

### 3.3. Secondary Outcomes

Of the studies, one did not report finding secondary outcomes [16]. In two RCTs [17,18], serum values of inflammatory markers and levels of stress-related hormones were investigated. In another article [20], the evaluation of the outcomes depends only on data obtained from scales or questionnaires based on pain, disability, psychosocial factors, and fall risk, through the NRS, TKS, RMDS, Patient-Specific Functional Scale (PSFS), and Falls Efficacy Scale (FES). In two of the studies included in the review, the secondary outcomes also included data on the results of exergames or the required activity with VR [5,19]. Stamm et al. [21] evaluated general physical and mental health (SF-12), but no significant changes were observed for either group. Interestingly, they also evaluated user experience and immersion (Technology Usage Inventory—TUI and User Experience Questionnaire—UEQ). Meinke et al. [22] evaluated the movement of the lumbar spine and hip, measured during two different movement tasks, the box lift and waiter bow tasks. In addition, they also assessed pain intensity, disability, quality of life, and fear of movement. However, no significant changes were observed in the lumbar spine and hip movements during the box lift and waiter bow tasks. Participant-reported outcomes (pain intensity, disability, quality of life, and fear of movement) did not show significant changes to the study’s hypotheses. As a result, Meinke et al. [22] reported the adherence rate, which was particularly high in the VR intervention group (55 out of 90, with a median of 61%). Mueller et al. [23] assessed, as secondary outcomes, angle reproduction, the maximum angle in secondary movement planes (trunk extension/flexion and rotation during lateral flexion), movement speed, and duration. These authors found that the upper trunk segment showed a significant decrease in the maximum angle for trunk extension/flexion from pre- to post-intervention (from 4.4° ± 4.4° to 3.5° ± 1.29°, *p* = 0.02, d = 0.20). These findings indicate that while the primary movement (lateral flexion) may not be immediately impacted by a single session, there may be positive acute effects on secondary movement planes, potentially improving trunk control in patients with CNS-LBP (see Table 4).

### 3.4. Technological Equipment

Two studies have a similar type of technology [17,18] consisting of a platform as a trunk movement sensor; in fact, the aim is to provide motor feedback to the patient on the activity of the core musculature and their balance competence and ability. Trunk movements are requested by the game through signals. In the study by Afzal et al. [16], the technology used is based on virtual reality exposure, 5′ for each type of game, consisting of motion-sensitive input embedded with a Time-of-Flight (TOF) sensor with gesture recognition and skeletal movements in real-time, all connected to an LCD screen. In the study by Matheve et al. [19], technology with wireless sensors placed at the sacral level (S2, for game input) and at the lumbar level (L1, for system calibration) is used. The game was played on a screen where audio was also maintained. Zadro et al. [20] are the only ones who uses a console (Nintendo Wii Fit U); the software and its parameters are standard and cannot be changed during the progression of the treatment. Thomas et al. [5] used an optoelectrical system, a technology that detects the movements made during the game. It comprises reflective markers on different landmarks on the head, arms, hands, chest, and pelvis. Stamm et al. [21] used an HTC Vive VR platform (head-mounted display plus controllers). In addition, these authors monitored patients’ strain with a real-time stress assessment through photoplethysmography. Detected changes in cardiac rhythm were categorized based on strain. The heart rate was shown in real time on the therapist’s interface, providing both information and control for the therapist. Kammler-Sücker et al. [24], as well as Afzal et al. [16], used the Kinect device to drive VR rehabilitation. In particular, Kammler-Sücker et al. [24] analyzed trunk movements (i.e., bending sideward and rotation in the horizontal plane). Alemanno et al. [25] used a VR therapy station, called a Virtual Reality Rehabilitation System (VRRS), connected to a six degrees of freedom tracking system (Polhemus G4 tracking system). Two sensors were placed on the manubrium of the sternum and the anterior superior iliac spine. In this way, the authors were able to collect kinematic data on the maximal and the average trunk’s range of motion during ten consecutive rotations, flexions, extensions, and lateral flexions. On the other hand, Mueller et al. [23] and Meinke et al. [22] used the Valedo Pro (Hokoma, Switzerland) for VR rehabilitation, which consists of two inertial measurement sensors, application-based software, and a tablet/smartphone. Thanks to the wearable sensors, the patient guides and controls his/her avatar through body movements. The two sensors were placed over the lower lumbar spine as well as the sternum during upright standing. Specifically, Mueller et al. [23] performed a kinematic analysis of trunk movements using a 16-camera optoelectronic 3D motion analysis system (Optitrack, Oregan, USA; 120 Hz). They placed ten markers on the torso, four markers around the pelvis, and an additional marker was positioned on the right scapula to enhance the identification of the left and right sides of the tracked skeleton. Differently from Mueller et al. [23], Meinke et al. [22] recorded center of pressure (COP) displacements during quiet standing on a stable force platform (AMTI, Accusway Plus).

### 3.5. Type of Intervention

In terms of exercise movements, the authors of the selected articles requested specific motor tasks in the VR environment (for more details, see Table 5). For example, Afzal et al. [16] administered a specific VR intervention protocol that included 5 min of trunk slide flexion, sitting to avoid obstacles, jumping, and combined arm movements. After a 30 s rest, a body ball game was introduced, which involved moving the arms, head pushing, and kicking the ball for an additional 5 min. The control group underwent routine physical therapy, which included a moist hot pack and hamstring stretching, along with back strengthening exercises. These exercises consisted of 10 repetitions each of bridging, prone leg raises, trunk extensions in a prone position with arms behind the back, trunk rotation exercises, knee-to-chest exercises, and diagonal elevation of the arm and leg in a prone position.

Matheve et al. [19] administered a single-session intervention consisting of two sets of 2 min of pelvic tilt exercises in the sagittal plane, with a 30 s rest in between. These exercises were designed to improve movement control of the lumbar spine and pelvis and were performed in a standing position with slightly bent knees. Participants placed their hands on their hips to guide pelvic movements. The control group also performed pelvic tilts in the sagittal plane, guided by a beep tone. Upon hearing the first beep, participants tilted their pelvis anteriorly and held the position until the next beep, after which they tilted it posteriorly, continuing this pattern. Participants completed 46 tilts during the first 2 min and 54 tilts during the second. In contrast, Zadro et al. [20] trained patients with Wii Fit U exercises for one hour, three times a week. However, they did not specify in detail the type of exercises performed by the patients.

Thomas et al. [5] trained patients using a specific virtual task involving a virtual dodgeball intervention. Patients were instructed to flex their lumbar spine, with lumbar flexion defined as the change in joint angle from the initial posture before each ball launched to the maximum joint angle during the trial. After each ball launch, patients were directed to return to an upright posture. The average gameplay session lasted approximately 15 min.

Nambi et al., (a) [17] as well as Nambi et al. (b) [18], trained patients in a VR group, focusing on the balance of stability of the core muscles for 30 min per session. The training was delivered in the sitting position, which challenged the balanced activities of the participants. Specifically, the virtual game was executed and controlled by moving the trunk back according to the signs. Participants performed all possible spinal movements within their pain limits. In addition, Nambi et al. (b) [18], administered isokinetic training, which consisted of isokinetic exercises for trunk extension and flexion, with the range of motion maintained between 10 degrees of extension and 80 degrees of flexion. Lastly, the control intervention was based on conventional core muscle-strengthening exercises focusing on the abdominal and back muscles, as well as stretching exercises for the hamstrings, hip flexors, and lumbar extensors. Exercises were performed 10–15 times per day, with stretching repeated three times for 10 s. In Nambi et al.’s study (a) [17], the authors introduced a combined physical therapy group that received conventional balance training using a Swiss ball (Fitness World, Italy) to target core muscles. The exercises included supine bridge, sit-ups, arm–leg cross lifts, and side bridges, performed in sets of 10 repetitions, three sets per session, five times a week for four weeks. Meanwhile, the control group followed conventional balance training, consisting of active, isotonic, and isometric exercises for the abdominal, deep abdominal, and back muscles.

Stamm et al. [21] implemented a specific 45 min exercise protocol for patients in the intervention group. This included warm-up exercises for the upper and lower extremities, strengthening of the abdominal and back muscles, and core stability training as the main component. The session concluded with stretching, progressive muscle relaxation, and a psycho-educational session delivered via a VR headset at the end of each training week. The control group followed the same exercise protocol for the same duration but without the use of VR technology.

Alemanno et al. [25], using the VRRS, trained patients with various motor exercises aimed at restoring a correct body image and improving trunk movement control. The one-hour exercises involved trunk rotation, flexion, and extension performed in different positions, including standing, sitting, and kneeling. Meinke et al. [22] administered ten VR postural exercises, which consisted of movements of the upper body or the pelvis. Trunk movements were performed on the sagittal, frontal, and transversal plane, and hip movements were performed on the sagittal and frontal plane. Participants could see on the display how well they matched the specified movement trajectory while playing, and further auditory feedback was provided. Additionally, Mueller et al. [26] introduced a VR exercise where patients imitated five trunk and pelvic movements, primarily focusing on right- and left-sided lateral flexion at three different levels. Each session lasted 12 min, with a resting phase of the same duration. Similarly, Kammler-Sücker et al. [24] administered exercises where patients imitated movements guided by VR technology. The exercises included lateral flexion of the spine, spinal rotation in the horizontal plane, and lifting a crate of water bottles, placing it on a chair, and then returning it to the floor. Participants were instructed to repeat each movement nine times per session.

## 4. Discussion

To the best of our knowledge, this is the first systematic review investigating the effects of sensor-based VR therapy in patients affected by CNS-LBP. Other authors [8] investigated the effectiveness of VR interventions in this patient population, without considering the use of specific sensors during training. Therefore, these authors [8] found that VR approaches can reduce pain intensity and kinesiophobia in patients with CLBP after the intervention and at the 6-month follow-up. These findings are in line with our results, suggesting that VR interventions could be a promising adjunctive treatment for patients with CNS-LBP. These results could be explained by the fact that VR interventions can easily catch the attention of the users, redirecting their cognitive focus from their bodies to virtual tasks, which can lead to pain reduction [27]. A simulated environment exploiting various postures, movements, or situations could assist therapists and patients in better understanding when and how pain occurs [28]. This simulation could display postures and movements from both first-person and third-person perspectives. In the first-person view, appropriate kinesthetic and proprioceptive devices would be crucial for effectively eliciting a sense of body ownership [28,29]. In the third-person view, observing another person or avatar would activate the mirror neuron system, which is essential for successful VR sensorimotor rehabilitation [28,29,30]. In addition, through VR, the triggering stimuli can be adapted to patients’ needs, varying their intensity, duration, repetition, and so on [28]. All these effects could increase patients’ awareness of their pain-related experiences and enhance pain management [28]. For instance, patients might better understand what triggers their pain, learn how to manage these triggers, and identify events that may alleviate their pain [28,31]. In this way, therapists could better understand their patients’ pain-related experiences. On the other hand, rehabilitation delivered by sensors has many other advantages, such as monitoring a patient’s motion and behavior during sessions in a controlled environment and providing audiovisual feedback via full-body immersion [32]. In this way, physiotherapists can adjust the level of difficulty, as well as the speed of exercise execution. By using these devices, physiotherapists can treat more than one patient simultaneously, thus reducing the costs of rehabilitation [32]. In the selected studies, the use of wearable sensors has led to an improvement greater than traditional rehabilitation. For example, depending on the type of exercise, it was shown that with VR, people could achieve better postural control; this change was not of a greater magnitude than the classical approach with an exercise program, but VR encouraged better performance. In all cases at the end of treatment, there were improvements in the outcomes considered compared to the baseline assessment. Even where the comparison concerns the magnitude of variation in outcomes within the groups, the experimental group had a greater tendency to improve than the others. Moreover, some authors [16,21] performed a combined approach, associating VR intervention with conventional physiotherapy or psychotherapy to enhance the effects of the VR training. Matheve et al. [7] pointed out that the application of standard exercises together with technology has a superior effect on CLBP than standard treatment alone. In two publications by Nambi et al., 2021 [17,18], exercises are combined with virtual reality treatment but are only prescribed at home. On the other hand, Stamm et al. [21] administered a combined approach, consisting of VR exercise therapy plus psychoeducation. These authors suggested that this multimodal approach was effective in promoting improvements in subjective functional capacity. However, no significance was found related to fear avoidance beliefs and general physical and mental health. Therefore, it is not possible to determine from this review how effective VR therapy is in combination with or in comparison with other approaches, both physical and psychological.

### 4.1. Sensor-Based Technology

In the rehabilitation context, IMUs can be used to collect motion data linked to the body segment where it is worn. By connecting multiple sensors to create a full-body model, joint movements can be deduced [32,33]. These devices can integrate several sensors, including an accelerometer, gyroscope, and magnetometer, enabling the implementation of robust sensor fusion algorithms to deliver accurate and detailed information across various dynamic conditions and applications [32]. In biomechanics, the primary application is estimating the device’s orientation using its embedded sensors to determine joint angles [34]. Additionally, IMU sensor data are utilized to analyze different aspects of human motion, with specialized algorithms developed for tasks like activity recognition and exercise recognition and evaluation [34]. Even if inertial sensors are one of the most used sensors in biomechanical evaluation, other methods can also be applied, such as stabilometric platforms, digital goniometers, pressure biofeedback, and optoelectronic systems [32]. Herrero and colleagues [35] investigated the use of sensors to assess patients with chronic NS-LBP in the context of personalized medicine. The authors [35] found that sensor systems can effectively identify certain characteristics indicative of CNS-LBP, thereby playing an important role in the diagnosis, prevention, and management of this condition. In particular, they selected studies involving both wearable devices, such as accelerometers, angle sensors, surface EMG, and IMUs, and non-wearable devices, such as force plate systems, a six-camera motion analysis system (Vicon), and the GAITRite mat with pressure sensors. In our systematic review, almost all of the articles included used inertial sensors or optoelectronic systems with advanced exergaming software that provides motion data analysis. The technologies that utilize movement sensors and augmented performance feedback can deliver reliable real-time feedback when combined with game-based tasks, making them advantageous for this therapeutic approach. Moreover, the cost of this technology can vary based on several factors such as the type of sensors used (e.g., tri-axial accelerometers or optoelectronic systems with markers), the number of sensors (e.g., a single sensor versus a full sensor suit), and the setup requirements. For example, optoelectronic systems require a specialized room and setup, along with trained personnel that increase the overall costs of the device. Among the selected studies, only Mueller et al. [23] used an optoelectronic system with 16 cameras and 3D motion analysis that has a high cost. In addition, Stamm et al. [21] used an HMD-VR platform, which is relatively low cost when compared with other VR devices or conventional MoCap. In fact, the HMD-VR platform exploits the conventional tracking methodology of these technologies by cutting down on costs and complexity, thanks to their link with the world of consumer electronics.

Previous studies have demonstrated the positive impact of sensor-based feedback over mirror- or therapist-based feedback. Matheve et al. [19] showed a hypoalgesia effect after just one session of game-based exercise intervention. However, the impact on trunk movement and motion control in patients with chronic low back pain (CLBP) is still uncertain. By using sensor-based technologies, therapists can customize the characteristics of the game according to the patient’s diagnosis, allowing a more personalized analysis of the feedback obtained [36]. Furthermore, audio/visual biofeedback or even tactile signals are emitted during the game, making the activity performed more real and engaging, as well as stimulating the game participant toward better motor adaptations [37]. In the case of Zadro [20], the use of a console as technology (Wii Fit U) was applied in an elderly population at home. In the study by Sims et al. [38], this technology was already being used, with positive results in its use for 2 weeks in patients with lower back problems. However, none of the included studies utilized electromyography (EMG) signals coupled with IMUs. For instance, some authors describe a method for remotely evaluating the characteristics of rehabilitative exercise that involves using sEMG sensors in addition to IMU sensors to monitor individuals during training. According to a previous review [35], patients affected by NS-LBP have a higher muscle activation in the posterior muscles (lumbar multifidus and erector spinae) than the healthy population. To this aim, a previous review [39] highlighted that this increased muscle activation could be related to different aspects, like (1) the limitation of movement and protection of sensitive, painful tissues; (2) compensation for muscle weakness caused by atrophy and fatty infiltration of the multifidus in response to back pain; or (3) alterations in proprioception.

### 4.2. Treatment Duration, Frequency, and Follow-Up

The articles included did not have uniform treatment periods and durations of sessions, preventing a shared proposal of these parameters with the use of the technology. In the analyzed articles, the treatment duration takes place mostly over approximately one month [16,17,18], where one publication proposes 2 months [20], another publication presents three consecutive days of treatment [5], and in one case, treatment takes place in a single session [19]. Regarding the duration of a single session, there is substantial variability. A meta-analysis [40] found the average time to be 8 weeks, with a total average amount of 12 h of treatment performed. In a meta-regression review on the treatment with core stabilization exercises in patients with NS-LBP [23], the greatest effectiveness was found with an average of three to five sessions per week; for a single session duration of approximately 20–30 min, improved outcomes in pain and disability were found. Most of the articles included focus on pain, disability, and psychosocial factors as both primary and secondary outcomes. A systematic review [41] found that LBP is linked to limitations in functional independence and participation in social activities. This highlights the need to investigate psychosocial factors, along with patients’ perceived disability, in rehabilitation studies. For example, Matheve et al. [19], as well as Thomas et al. [5], assessed different aspects of pain and disability in people with LBP. In addition, Matheve et al. [19] provided results about patients’ motivation, since one of the most important barriers in rehabilitation is adherence to the training program, especially at home.

Moreover, the emotional and behavioral components are more and more involved in the assessment; in fact, the experience of pain and impairment related to motor difficulties with NS-LBP and CLBP. The most widely used scale is the TKS [18,19,20] followed by the PCS [5]. Previous studies [42,43,44] have explored the correlation between psychological comorbidities such as anxiety, depression, and somatization symptoms in patients with CLBP. In this context, depression and anxiety often act as barriers to treatment adherence, preventing the success of rehabilitation efforts. Therefore, from a clinical perspective, it is crucial to assess the psychological impact of CLBP to enhance the overall effectiveness of rehabilitation interventions. Multidisciplinary biopsychosocial rehabilitation interventions are more effective than standard care and physical therapy alone in reducing pain and disability in individuals with chronic low back pain [22]. This is particularly relevant for the elderly, where a reduced load-bearing capacity and impaired body sway make them more vulnerable to issues with stability and balance [36]. In this context, technology plays a crucial role not only in providing preventive analysis but also in delivering active treatment to reduce the risk of falls and enhance overall rehabilitation outcomes [36]. In this context, it may be beneficial to assess sensory impairments that could potentially interfere with VR rehabilitation, particularly in elderly patients. However, only Stamm et al. [21] considered this important factor when enrolling participants. They excluded patients with conditions such as dizziness or severe visual impairments (e.g., oscillopsia), which could negatively impact VR training.

Furthermore, cognitive tasks within a motor-type exercise make it more challenging to perform because it becomes a ‘dual task’ [45]. There are two related factors, the demand for ‘action-ready’ cognitive functions that are closely linked to learning a motor act such as attention, concentration, and memory; and the fact that motor control uses cognitive resources to be performed [45]. In contrast, reduced performance at the cognitive level may also result in reduced motor performance; this may expose one to the persistence or multiple occurrences of LBP or the development of CLBP.

Another aspect that is related to pathophysiological is changes in body muscles in patients with NS-LBP. In fact, it could be useful to have results related to the correlation of variation in muscle volume and physical activity with NS-LBP to also assess the efficacy of the rehabilitation. However, only Nambi et al. [18] 2021 specifically addresses muscle volume change as a treatment goal.

## 5. Limitations

The study’s main limitations were the different methodological approaches in terms of the type of game, duration, type of technology, and the positioning of the sensors. The necessity of being able to compare clinical parameters more objectively would provide a clearer view of the usefulness and applicability of exergaming and biofeedback training in clinical practice. It is also necessary to develop a common line of approach that may be effective, easy to apply, and can be adopted by different age groups.

Moreover, this systematic review has some limitations too, primarily related to the lack of a quantitative analysis. We did not perform a quantitative meta-analysis since we found great heterogeneity among the included studies that can make statistical synthesis inappropriate or unreliable. In addition, the included studies have small sample sizes that may not provide enough statistical power for meaningful synthesis in a meta-analysis, leading to unreliable or inconclusive results. Lastly, we also found great heterogeneity in the outcome measures used to assess patients with LBP. Despite these limitations, our review relies mainly on qualitative synthesis, based on systematically summarizing and interpreting the findings of individual studies to elucidate common themes, patterns, and discrepancies across the literature. As a result, our review provided a comprehensive qualitative synthesis of the available evidence, offering valuable insights into the innovative sensor-based plus VR rehabilitation approach for patients affected by CNS-LBP, identifying key implications for clinical practice and considerations for future investigation.

## 6. Conclusions

This systematic review reported that VR rehabilitation therapy, augmented by sensor-based technology, shows significant potential as an adjunctive intervention for patients with CNS-LBP. The findings suggest that such interventions, particularly through exergames, can effectively reduce pain and disability while also addressing psychosocial factors such as kinesiophobia. Importantly, the use of IMUs and other motion sensors provides valuable biofeedback, enhancing patient engagement and treatment adherence—crucial elements for successful rehabilitation outcomes.

Despite the promising results, there remains variability in treatment protocols, session duration, and follow-up across the studies reviewed, which limits the ability to derive a standardized approach. The integration of VR and sensor-based technologies into clinical practice should therefore be further investigated with the goal of optimizing protocols and ensuring broader applicability across different patient populations and clinical settings. Additionally, while this review confirms the potential efficacy of these technologies in controlled environments, their effectiveness in real-world clinical practice, especially over the long term, requires further validation through larger studies.

Ultimately, sensor-based VR rehabilitation presents a novel, non-pharmacological approach that may complement traditional therapies for CNS-LBP, especially in enhancing motivation and adherence to exercise regimes. Future research should focus on establishing consistent treatment guidelines, assessing cost effectiveness, and exploring the long-term impact on patients’ quality of life.

## Figures and Tables

**Table 1:** Search strategy used for the selection of the studies.

Database	Search Query	Filters
PubMed	((((inertial measurement unit) OR (sensor-based) AND (virtual reality)) OR (exergame)) AND (low back pain))	No filters
Scopus	inertial measurement unit OR sensor-based AND virtual reality OR exergame AND low back pain	Range time 2016–2024; Articles
Science Direct	inertial measurement unit OR sensor-based AND virtual reality OR exergame AND low back pain	No filters
WoS	inertial measurement unit OR sensor-based AND virtual reality OR exergame AND low back pain	Range time 2016–2024; Articles

**Table 2:** Summary of the characteristics of the studies included in the systematic review.

	Type	Groups	Exercise Group
Afzal et al., 2022 [16]	Single-blind RCT	42 exercise group + 42 control group	VR exergame + physical therapy
Nambi et al., 2021 (a) [17]	Single-blind RCT	12 VR group + 12 combined group + 12 control group	VR exergame
Nambi et al., 2021 (b) [18]	Double-blind RCT	20 VR group + 20 isokinetic group + 20 control group	VR exergame
Matheve et al., 2020 [19]	RCT	42 VR group + 42 control group 30 exergame group + 30 control group	VR exergame
Zadro et al., 2019 [20]	RCT	26 VR group + 26 control group	Exergame
Thomas et al., 2016 [5]	RCT	11 intervention group + 11 control group	VR exergame
Stamm et al., 2022 [21]	RCT	10 VR intervention + 10 control group	VR therapy (physical therapy + psychoeducation)
Meinke et al., 2022 [22]	Pilot RCT	20 VR treatment	VR exergame
Alemanno et al., 2019 [25]	Proof-of-concept study	13 VR rehabilitation + 14 control group	VR therapy
Kammler-Sücker et al., 2023 [24]	Feasibility study	Imitated an avatar (AVA) N = 17 + videotaped model (VID) N= 16	Virtual doppelganger avatars in the virtual environment
Mueller et al., 2022 [26]	Randomized cross-over pilot trial	13 VR intervention and control	Game-based real-time feedback intervention

**Table 3:** Quality assessment of included studies using the PEDro scale [13].

	PEDro Items	Afzal et al., 2022 [16]	Nambi et al., 2021 [17]	Nambi et al., 2021 [18]	Matheve et al., 2020 [19]	Zadro et al., 2019 [20]	Thomas et al., 2016 [5]	Stamm et al., 2022 [21]	Meinke et al., 2022 [22]	Alemanno et al., 2019 [25]	Muller et al., 2022 [26]	Kammler-Sücker et al., 2023 [24]
1	Eligibility criteria	1	1	1	1	1	1	1	1	1	1	1
2	Random allocation	1	1	1	1	1	1	1	1	0	1	1
3	Concealed allocation	1	1	1	1	1	1	0	0	0	1	0
4	Baseline similarity	1	1	1	1	1	1	1	1	0	1	1
5	Blinding of subjects	0	0	1	0	0	0	0	0	0	0	0
6	Blinding of therapists	0	0	0	0	0	0	0	0	0	0	0
7	Blinding of assessors	1	1	1	1	1	1	0	1	0	0	0
8	Measures of key outcomes from more than 85% of subjects	1	0	1	1	0	0	0	0	1	1	1
9	Intention to treat analysis	1	1	1	1	1	1	1	1	0	0	0
10	Between-group statistical comparisons	1	1	1	1	1	1	1	1	0	1	1
11	Point measures and measures of variability	1	1	1	1	1	1	1	1	1	1	1
	Total	8	7	9	8	7	7	6	7	3	7	6

**Table 4:** Characteristics of assessment type, primary and secondary outcomes.

	Assessment: Baseline End Treatment/Follow-Up	Primary Outcome	Secondary Outcome
Afzal et al., 2022 [16]	Baseline revaluations: 4th/8th/12th session	VAS, MODI	_
Nambi et al., 2021 (a) [17]	Baseline + evaluation at the end of treatment (4th week)	CSA with RMN + muscle thickness with US (paraspinal ms.)	Inflammatory biochemical parameters
Nambi et al., 2021 (b) [18]	Baseline + evaluation at the end of treatment (4 weeks) + follow-up at 6 months	VAS 0–10 TSK	Serum level of stress-related hormones
Matheve et al., 2020 [19]	Baseline + evaluation at the end of the treatment (single session)	Difference in pain between baseline and during exercise NPRS, PCS, TSK, RMDS	Difference in pain between baseline and immediately after exercises + time spent thinking about pain during exercises + no. of pelvic tilts performed + motivation for movement and perception of harmfulness
Zadro et al., 2019 [20]	Baseline evaluation at 8 weeks (end of treatment) + follow-up at 3/6 months	PSEQ Care seeking RAPA data collected at 8 weeks, 3–6 months	NRS, PSFS, RMDQ, TSK, FES-I (data from these scales collected at 8 weeks) treatment adherence, treatment experience adverse events
Thomas et al., 2016 [5]	Baseline + follow-up on day 4 or 6. Only in pre-treatment: also STAI	Expectation of pain or damage: VAS degree of trunk flexion with 3D software	Degree of trunk flexion during exergaming: different levels with successive flexion increments and return to starting position + game experience survey, current pain, and medication RMDQ, MPQ
Stamm et al., 2022 [21]	Baseline and anamnesis and interview, orthopedic examination + evaluation at the end of the treatment (4 weeks, with 3 appointments lasting approximately 30 min)	NRS, CPGQ, Ffb-H-R, TSK-11	TUI, SF-12
Meinke et al., 2022 [22]	Outcomes were evaluated at two points, initially at T1 and T2 before any intervention, followed by assessments at T3 after a fixed 3-week exercise regimen for the intervention group and at T4, which occurred 6 weeks later without a specified exercise schedule	Postural assessment (CoP) and movement task with IMUs, NRS, RMDQ, WHOQOL-Bref, TSK-11	As secondary outcomes, the movement of the lumbar spine and hip during 2 different movement tasks and participant-reported outcomes were included
Alemanno et al., 2019 [25]	Before and after treatment, subjects underwent a comprehensive neuropsychological assessment and a physical therapy examination	ADL, IADL, MMSE, Attentive and Raven Matrices, Token test, semantic fluency, phonemic fluency and naming, word picture matching test, digit span test, Digit Span Backward, Corsi block-tapping test, Rey Complex Figure Test, Trail making test, Stroop test, Wisconsin Card Sorting test, BDI, SF-36, NRS, MPQ, BPI, RMDQ, and kinematic data were measured using the Polhemus G4 tracking system, measuring the maximal and the average trunk’s range of motion during ten consecutive rotations, flexions, extensions, and lateral flexions	
Kammler-Sücker et al., 2023 [24]	Participants answered questions at three time points during the experimental sessions; in the beginning, after the movements when still in the virtual environment, and after having left the virtual environment	FABQ, MPI1, NRS, GCPS, HADS, FFbH, FABQ	
Mueller et al., 2022 [23]	After receiving an anthropometric (body height (cm) and body mass (kg)) assessment, all participants completed a paper–pencil-based version of the graded chronic pain questionnaire (von Korff), valid to measure the presence of chronic low back pain	Maximum angle (MA°) during left- and right-sided maximum lateral flexion movement while upright standing	Movement velocity (°/s) and duration (s), movement cycle from upright standing to maximum (left- or right-sided) lateral flexion and back to upright standing. Graded pain questionnaire, VAS

Legend: MODI (Modified Oswestry Disability Index), CSA (cross-sectional area section), TSK (Tampa Scale for Kinesiophobia), RMDQ (Roland Morris Disability Questionnaire), PCS (Pain Catastrophizing Scale), NPRS (Numeric Pain Rating Scale), PSFS (Patient-Specific Functional Scale), FES-I (Falls Efficacy Scale International), PSEQ (Pain Self-Efficacy Questionnaire), RAPA (Rapid Assessment for Physical Activity), STAI (State-Trait Anxiety Inventory), CPGQ (Chronic Pain Grade Questionnaire), Hannover Functional Ability Questionnaire for measuring back pain-related disability (Ffb-H-R); Tampa Scale of Kinesiophobia (TSK-11); SF-12; Technology Usage Inventory (TUI); Roland Morris Disability Questionnaire (RMDQ); The World Health Organization Quality of Life Questionnaire—short version (WHOQOL-Bref); Activities of Daily Living (ADLs); Instrumental Activities of Daily Living (IADLs); Mini Mental State Examination (MMSE); Attentive and Raven Matrices; Token test; Beck Depression Inventory-II (BDI); SF36—Short-Form Health Survey; McGill Pain Questionnaire (MPQ); Brief Pain Inventory (short form) (BPI); Multidimensional Pain Inventory (MPI1); Graded Chronic Pain Scale (GCPS); Hospital Anxiety and Depression Scale (HADS).

**Table 5:** Summary of technology applied and VR treatments.

	Technology Description Software Details	Treatment Detail in VR: Exercises + Frequency/Treatment Duration	Results: Variations on Outcomes
Afzal et al., 2022 (RCT) [16]	Kinetic device, where motion input is incorporated with Time-of-Flight sensor.	Ball and reflex game (reflex ridge); duration of sessions 25 min, with 5 VR+ 20 RPT; total 12 sessions, 3 sessions/week.	Intra-group: in both experimental and control group, ↑ of VAS and MODI. Change in pain and disability pre-post is >per experimental group. In particular, pain score at baseline was similar for both groups (RPT and VR game, respectively) 6.62 + 1.04 and 6.50 + 1.24, which decreased to 3.32 + 0.81 and 1.00 + 0.60, respectively, after the 12th session (*p* < 0.05). In addition, functional disability score at baseline was 65.08 + 8.94 in RPT group and 69.16 + 9.13 in VR game group, which decreased to 40.56 + 8.59 and 16.04 + 6.82, respectively, after the 12th session (*p* < 0.05).
Nambi et al., 2021 (a) [17]	Platform connected to a screen (ProKinsystem, PK252,NTeco, Body, Italy).	Game based on balance and core stability in a seated position. Duration 30 min, 5 sessions/week for 4 weeks.	Regarding CSA with MRI, ↑ pre- and post-treatment with VRT group compared to other groups; with CPR and control < . CSA measured with US (multifidus), same result. Inflammatory biomarkers, all 3 groups ↑ pre- and post-treatment, but experimental group >. Specifically, the VRT group showed more significant changes in the muscle CSA, rather than control group and CPR (*p* < 0.001). In addition, several biochemical measures (including tumor necrosis factor and interleukines) also showed significant improvement in the VRT group compared to the other two groups (*p* < 0.001).
Nambi et al., 2021 (b) [18]	The patient is seated on a virtual platform displayed on a screen (ProKinsystem, PK252,NTeco, Body, Italy).	Game based on balance and stability of the core muscles, in a seated position. Duration 30 min, 5 sessions/week for 4 weeks.	VAS: EG and the other two groups ↓ at the end of treatment/follow up; however, in the groups with virtual reality and isokinetics, >. TKS: post intervention > with virtual reality and isokinetics. Serum hormone levels: EG and 2 CG ↑ at 4 weeks; at 6 months glucose/insulin =; the remaining parameters ↑. With glucose/insulin EG > vs. 2 CG. In detail, VRT and isokinetic groups showed significant changes in pain intensity and kinesiophobia in comparison to the control group (*p* < 0.05). Hormonal measures also showed significant improvement in the VRT group in comparison to the other two groups (*p* < 0.05).
Matheve et al., 2020 [19]	Wiereless motion sensors (Valedo Home; Hocoma, Switzerland), placed with double-sided tape on S2 (+ 1 sensor used for calibration on L1). Measurement accuracy is 0.1° at 50 Hz frequency.	Game based on pelvic tilt movement; the patient is in standing position. Treatment in a single session with 2 repetitions of 2 min interspersed with a 30 s break.	During EG exercises, there was greater pain reduction and less time spent thinking about pain. Pain-related fear/catastrophizing, ↑ pre-/post-treatment intergroup level; = intragroup in EG and CG. EG motivation and perception of little harm (harmfulness). In particular, VR distraction had a hypoalgesic effect during (Cohen’s d = 1.29) and immediately after (Cohen’s d = 0.85) the exercises, and it also reduced the time spent thinking about pain (Cohen’s d = 1.31).
Zadro et al., 2019 [20]	Nintendo Wii U technology and Wii-Fit-U software (Redmond, WA, USA). The treatment was home-based.	Patients played games related to physical activity, such as yoga (5′), strengthening exercises (25′), aerobic exercises (10′), and balance exercises (20′); the latter are performed in virtual reality. Total treatment 60 min, 3 times/week, for 8 weeks.	PSEQ ↑ at 6-month follow-up (in comparison with CG), but not at the immediate end of treatment nor at 3-month follow-up. NRS and PSFS ↑ in the immediate post-treatment. Intergroup = disability, fear of movement/re injury, falls efficacy, care seeking, physical activity at 8 weeks and 3 months. Showed willingness to perform flexibility movements at least 1 time/week after 6-month follow-up. Adherence was complete but showed uneven distribution. Patients reported positive experience data; occasional symptoms were experienced at the end of the exercise session. Participants in the video game exercise group had significantly higher pain self-efficacy scores at 6 months compared to the control group (adjusted between-group difference: β = 5.17, 95% CI = 0.52–9.82, *p* = 0.03). However, there were no significant differences immediately post-intervention or at 3 months. In addition, participants in the intervention group reported significantly greater reductions in pain immediately post-intervention (adjusted between-group difference: β = −1.07, 95% CI = −2.11 to −0.03, *p* = 0.04). The intervention group also showed significantly greater improvement in function post-intervention (adjusted between-group difference: β = 1.21, 95% CI = 0.10–2.33, *p* = 0.03).
Thomas et al., 2016 [5]	Motion sensors on the head, arm, forearm, hand, trunk, pelvis, thigh, shin, and foot. Both the experiment (exergame) (WorldViz™) and the basic test are performed.	The game is developed with Vizard software. The patient has to play dodgeball against 4 virtual opponents. The treatment consisted of 3 consecutive 15 min days of exergames with dodgeball.	Primary outcome: between the 2 groups at baseline and post-treatment for lumbar flexion and hazard expectancy. Secondary outcome: lumbar flexion ↑ as an outcome of playing levels and impact heights. Follow-up analyses show ↑ of lumbar flexion at each game level. Game experience survey scores/comments: acceptability with high scores of liking reported by patients. No significant effects of group (F(1,50) = 1.96, *p* = 0.16), day (F(1,50) = 2.24, *p* = 0.14), or group by day interaction (F(1,50) = 0.21, *p* = 0.64) for lumbar spine flexion. A significant effect of day for expected pain (F(1,50) = 11.91, *p* = 0.001, ηp^2^ = 0.19) but no significant effects of group (F(1,50) = 1.25, *p* = 0.26) or group by day interaction (F(1,50) = 0.16, *p* = 0.68). Secondary Outcome: Significant main effects of game level (F(2,22) = 6.54, *p* < 0.01, ηp^2^ = 0.37) and impact height (F(4,20) = 33.3, *p* < 0.001, ηp^2^ = 0.87), but not day (F(2,22) = 1.45, *p* = 0.25). A significant day by impact height interaction (F(8,16) = 7.56, *p* < 0.001, ηp^2^ = 0.79).
Stamm et al., 2022 [21]	HTC Vive VR system (HTC Vive, HTC Europe Co., Ltd., Slough, Berkshire, UK) (consisting of VR headset and two controllers) and a laptop were used.	The developed VR game was composed of two software interfaces, a therapist interface and the VR game, in which the participant performs interactive tasks on a farm (e.g., rowing, turning on light bulbs, or sorting vegetables).	Only a significant improvement in the subjective functional capacity (Ffb-H-R) was achieved after the completion of a four-week multimodal pain therapy in VR. The VR therapy did not reach the pain intensity reduction in the CG (IG: MD = 0.64; CG: MD = 1.64). The functional capacity in the IG improved from Visit 1, x = 73.11% to Visit 2, x = 81.82% (MD = 8.71%; *p*= 0.026).
Kammler-Sücker et al., 2023 [24]	Kinect Sensor, (Microsoft, Redmond, WA) + motion capture with an infrared 12-camera system, (OptiTrack, Corvallis, OR, USA).	The exercises included lateral spine flexion (“bending sideward”), spinal rotation in the horizontal plane, and picking up a crate of water bottles (13 kg) to place it on a chair and then back on the floor (“crate-moving”). This procedure was repeated for all three movement types, with three repetition cycles for the entire sequence, and the order of movement types was randomized between cycles.	Regarding motor performance as measured by the range of motion (ROM), no significant differences were found between the groups, except for rotation in the horizontal plane (RH). For bending sideward (BS), there was no significant group effect or trend. Similarly, self-reports on pain and function after the movements showed no significant differences.
Alemanno et al., 2019 [24]	VRRS (Khymeia, Padua, Italy) + computer workstation connected to 6 degrees of freedom (DOF) motion-tracking system (Polhemus G4, Vermont, US), high-resolution LCD displaying the virtual scenarios on a large screen and software processing the motion data.	The aim of the exercises was to regain a correct body image by improving the control of single movements of the trunk. Patients underwent a series of exercises consisting mainly in trunk rotation, flexion, and extension in various positions (standing, sitting, and kneeling).	After six weeks of treatment, all pain scores showed significant reductions, with some exceeding the minimum clinically important difference noted in the literature. This reduction in pain was also accompanied by enhancements in quality of life, certain cognitive functions, and sensorimotor output. The data showed significant reductions in all pain rating scale scores (*p* < 0.05); significant improvements in quality of life in the domains of physical functioning, physical role functioning, bodily pain, vitality, and social role functioning; improvements in cognitive functions (*p* < 0.05); improvements in functional scales (*p* < 0.05) and mood (*p* = 0.04).
Meinke et al., 2022 [22]	The assessments were conducted using the Valedo Pro (Valedo Home; Hocoma, Switzerland), which includes three inertial measurement units (IMUs) and dedicated software. The IMUs were attached with medical adhesive strips at the height of the spinal process of the S1 and L1 vertebrae, and one IMU was placed on the left leg, 20 cm from the lateral femoral condyle.	Participants in the intervention perform upper body and pelvic movements across multiple planes (sagittal, frontal, and transversal for the trunk; sagittal and frontal for the hips). They receive real-time visual feedback on their movement accuracy displayed on a screen, supplemented by auditory cues. The session concludes with a performance ranking that compares their current and previous game outcomes.	Over a span of about 3 weeks, unsupervised home exercises focusing on trunk movements did not improve postural balance in participants with LBP or show significant effects on other measured outcomes. Comparisons between groups indicated a trend toward slightly increased lumbar spine motion during both tasks in the intervention group, contrary to expectations, while the control group showed a slight reduction. Adherence to the exercise program schedule was low, and after the schedule ended, only a few participants continued using the training device regularly without specific guidance. The intention-to-treat analysis found no significant differences in the change in anterior–posterior sway direction between the groups during the intervention period with the specified schedule (T2–T3) (W = 99; *p* = 0.36; r = 0.07). None of the outcomes aligned with authors’ hypotheses showed a significant change. Participants in the intervention group completed a median of 61% (55/90; range 2−99%) of the exercises in the prescribed training program, with higher adherence observed during the first intervention period under the specified schedule.
Mueller et al., 2022 [23]	The game-based real-time biofeedback training was conducted using a medical device designed for digital, home-based back pain therapy (Valedo Home; Hocoma, Switzerland). This system includes two inertial measurement sensors, application-based software, and a tablet or smartphone.	The intervention included a one-minute trunk stabilization task, a magic mirror task with five trunk/pelvis movements, and a movement game focused on lateral flexion through three levels. The entire session lasted 12 min, with an equivalent resting period.	A single session of game-based real-time feedback intervention resulted in changes in secondary movement planes, indicating reduced evasive motion during trunk movements in patients with chronic low back pain. No significant changes were observed in maximum angle or angle reproduction from pre- to post-intervention for any segment in the primary movement plane and lateral flexion (*p* > 0.05). However, the upper trunk segment showed a significant reduction in MA for trunk extension/flexion from pre- to post-intervention (4.4° ± 4.4° (95% CI 7.06–1.75) to 3.5° ± 1.29° (95% CI 6.22–0.80); *p* = 0.02, d = 0.20).

Legend: VAS (Visual Analogical Scale), MODI (Modified Oswestry Disability Index), RPT (routine physical therapy), CSA (cross-sectional area), MRI (magnetic resonance imaging), CPR (C-reactive protein), TKS (Tampa Scale of Kinesiophobia), PSEQ (Pain Self-Efficacy Questionnaire), NRS (numerical rating scale), PSFS, CI (confidence interval), Ffb-H-R (Hannover Functional Ability Questionnaire for measuring back pain-related disability), MA (maximum angle).

## Data Availability

Not applicable.
